# Antimicrobial Resistance and Stewardship in Surgical Departments: A Three-Year Single-Center Study

**DOI:** 10.3390/medicina62091756

**Published:** 2026-09-11

**Authors:** Adriana Grindean, Mihaela Elvira Cîmpianu, Elena Maria Domsa, Adrian Popentiu

**Affiliations:** 1 Department of Nursing, “1 Decembrie 1918” University of Alba Iulia, 510009 Alba Iulia, Romania; adriana.grindean@uab.ro (A.G.); maria.domsa@uab.ro (E.M.D.); 2 Department of Surgery, “Dr. Alexandru Augustin” Military Emergency Clinical Hospital, 550024 Sibiu, Romania; adipopentiu@yahoo.com; 3 Faculty of Medicine, Lucian Blaga University of Sibiu, 550169 Sibiu, Romania

**Keywords:** antimicrobial resistance, multidrug-resistant infections, antimicrobial stewardship, healthcare-associated infections, patient safety

## Abstract

*Background and Objectives*: Antimicrobial resistance represents an important patient-safety concern in surgical departments, where empirical therapy, invasive procedures, intensive-care exposure, and healthcare-associated infections may contribute to adverse outcomes. This study aimed to describe temporal changes in institutional surveillance indicators related to microbiological testing coverage, selected antimicrobial-resistance phenotypes among clinical isolates, selected-antibiotic use profiles, and infection-related patient-safety outcomes in surgical departments and the intensive care unit of a Romanian tertiary clinical emergency hospital. *Materials and Methods*: A retrospective, longitudinal, single-center observational study was conducted from January 2023 to December 2025. Data were extracted from four institutional sources: microbiology laboratory records, pharmacy antimicrobial-use data, the infection prevention and control registry, and the clinical-administrative hospital information system. Microbiological testing coverage, the selected resistance phenotypes and healthcare-associated infection indicators were analyzed at semester level, with selected-antibiotic DDD profiles being analyzed at department level. Statistical analyses included descriptive statistics, Cochran–Armitage trend testing, Fisher’s exact test, and exploratory Spearman correlation. *Results*: The hospital-wide microbiological testing intensity index based on 1892 antibiogram events among 8141 antibiotic-treated patient records increased significantly from 18.05% (Semester I 2023) to 28.20% (Semester II 2025). When expressed as proportions of clinical isolates, *E. coli* MDR decreased from 10/136 isolates (7.4%) in Semester II 2023 to 0/122 isolates (0.0%) in Semester II 2025, and *S. aureus* MRSA+MLSB decreased from 11/36 isolates (30.6%) to 0/22 isolates (0.0%) over the same interval. Undetected several semesters, *P. aeruginosa* XDR/PDR accounted for 4/25 clinical isolates (16.0%) in the final semester. Selected-antibiotic DDD profiles differed across departments, with General Surgery and ICU accounting for the largest selected-antibiotic totals. Preoperative bacteriological screening increased from 48 tests in Semester I 2024 to 329 tests in Semester II 2025, MRSA-positive screening results decreasing from 10/48 (20.8%) to 2/329 (0.6%) over this interval, this finding being interpreted descriptively because the screened population and screening protocol could not be fully standardized retrospectively. Among 46 validated cases of healthcare or *Clostridioides difficile* infections, the overall mean length of stay was 15.35 days, peaking at 20.80 days in surgical departments. *Conclusions*: Routinely collected hospital data can generate useful institutional surveillance signals related to antimicrobial resistance, antimicrobial use, and infection-related patient safety. Increased microbiological testing coverage and lower proportions of selected MDR/MRSA phenotypes were observed during the study period, while late detection of XDR/PDR *P. aeruginosa* highlighted the need for sustained microbiological surveillance and department-specific stewardship review.

## 1. Introduction

The global challenge of antimicrobial resistance (AMR) is a major health threat worldwide, with the emergence and unprecedented spread of multidrug-resistant organisms in the past two decades creating a significant public health crisis [1]. Since their discovery a century ago, antibiotics have revolutionized modern medicine, having saved millions of lives [2]. They play a vital role in surgical operations, being prescribed either as treatment or for prophylactic purposes to prevent surgical site infections (SSIs) [3]. A major cause of morbidity and mortality worldwide, SSIs are the most prevalent kind of healthcare-associated infection (HAI) in low- and middle-income countries [4]. It is estimated that HAI incidence is up to 20 times higher in low-income countries compared to high-income ones [5]. Annually endangering the lives of millions of people, SSIs fuel the occurrence of bacterial-resistant strains [6].

Systematic reviews have identified empirical prescribing practices, limited access to microbiological diagnostics, and unrestricted availability of antibiotics as contributing factors that facilitate AMR [7,8]. Bacterial infections caused by multidrug-resistant (MDR) and extensively drug-resistant (XDR) organisms are significantly linked to increased mortality and long-term disability, being directly responsible for over 1 million deaths in 2019 [9].

Identified decades ago as critical multidrug-resistant bacteria for which effective therapies were rapidly needed, the so-called ESKAPE pathogens (*E. faecium*, *S. aureus*, *K. pneumoniae*, *A. baumannii*, *P. aeruginosa* and *Enterobacter* spp.) continue to represent major therapeutic challenges as they share survival adaptations in the modern healthcare setting, acquiring resistance determinants to successfully disseminate worldwide [10]. These pathogens were designated by the World Health Organization (WHO) as critical or high-priority organisms due to their extensive resistance profiles. The WHO Global Antimicrobial Resistance and Use Surveillance System (GLASS) 2025 report highlights antibiotic resistance prevalence and trends based on 93 infection type–pathogen–antibiotic combinations in over 23 million bacteriologically confirmed cases of bloodstream infections, urinary tract infections, gastrointestinal infections, and urogenital gonorrhea reported in 110 countries between 2016 and 2023. The report presents adjusted national AMR estimates for 2023 for key pathogen–antibiotic combinations, tracking global and regional resistance trends between 2018 and 2023, and reviews progress in building global and national AMR surveillance systems since 2016. Based on these findings, the report proposes several recommendations to strengthen surveillance systems and support country-level responses aligned with the global targets [11].

European surveillance data corroborates these findings, elevated AMR levels across European Union countries being reported in relation to Enterobacterales resistance against third-generation cephalosporin and carbapenem, or regarding the high prevalence of carbapenem-resistant *Acinetobacter* spp. [12]. In order to enhance the detection and monitoring of carbapenemase-producing Enterobacterales, the European Centre for Disease Prevention and Control (ECDC) has implemented the CRE25 genomic surveillance protocol that integrates whole-genome sequencing (WGS) into routine AMR surveillance across Europe to help elucidate genomic organization, virulence potential, and host adaptation in bacterial pathogens [13].

A review on antimicrobial resistance commissioned by the British government produced an extremely disturbing scenario, predicting a yearly toll of 10 million AMR-related deaths and a financial burden of 100 trillion USD unless vigorous prevention policies were to be imposed. The report stated that 13 out of 40 million people on antimicrobial medication actually didn’t need them, while, for those in real need, antibiotics were not available. Four interventions were seen to be particularly important: a global public awareness campaign to educate about the problem of drug resistance, a policy rewarding the development of new drugs to replace inefficient ones, a limitation of unnecessary use favoring AMR (the availability of rapid point-of-care diagnostic tests being seen as the best solution to limit unnecessary antibiotic use), and a policy of banning or restricting the veterinary usage of vital human antibiotics [14].

Revealing that the health burden of bacterial AMR was equivalent to that of influenza, tuberculosis and HIV/AIDS combined, the Roadmap on antimicrobial resistance for the WHO European Region [15] called for political commitment and massive investments in new drugs.

Having recorded in 2021 the highest consumption of antibiotics per capita in the European Union (25.7 DDD/1000 inhabitants/day), Romania has developed the National Strategy 2023–2030 for the prevention and limitation of healthcare-associated infections, setting a series of AMR targets for 2030: to reduce by 27% the total consumption of antibiotics in humans (compared to 2019), to ensure that at least 65% of the total consumption of antibiotics in humans belongs to the ‘Access’ group of antibiotics as defined in the AWaRe (Access/Watch/Reserve) classification of the WHO (+12.2% compared to 2019), to reduce by 18% the total incidence of bloodstream infections with meticillin-resistant *S. aureus* (MRSA) and by 5% the total incidence of bloodstream infections with third-generation cephalosporin-resistant *E. coli*, as well as that with carbapenem-resistant *K. pneumonia*, per 100,000 population [16]. These actually outbid the European Union’s 2030 targets, set to reduce antibiotic consumption by 20% and MRSA infections by 15% [17].

According to the ECDC’s 2023 Epidemiological Report, reaching all these AMR targets by 2030 will be impossible in the absence of stronger public health action, the consequence being an increased number of infections with antibiotic-resistant bacteria that will ultimately lead to an increased number of AMR-related deaths. Even more so in case of Romania, the only EU member state with a low geographical, hospital and isolate representativeness over the 2018–2023 period, as stated in the latest ECDC Annual Epidemiological Report upon data communicated by RO-RNSSy, partner in The European Surveillance System (TESSy) platform designed for the collection, analysis and dissemination of data on communicable diseases. While in Denmark, Estonia, Iceland, Lithuania or Luxembourg the estimated population coverage was 100% (EU average 75.4%), in Romania it was 13% [18].

Intervention strategies addressing the challenge of bacterial AMR need to take into account basic prevention principles, including both hospital-based infection prevention and control programs focused on preventing healthcare-associated infections and community-based programs focused on water, sanitation, and hygiene. Because the present study integrates antimicrobial-resistance indicators, microbiological surveillance, antimicrobial-use data, and stewardship-related outcomes, transparent reporting according to recommendations for epidemiological studies on antimicrobial resistance and antimicrobial stewardship is essential [19]. Antibiotic stewardship is a key instrument in preventing the spread of AMR. While it generally implies limiting access to antibiotics, in some cases (e.g., western sub-Saharan Africa) it may imply granting access to necessary antibiotics. Developing vaccines for all ESKAPE pathogens and reducing exposure to antibiotics unrelated to the treatment of human disease are important targets, as is minimizing the unnecessary use of antibiotics (e.g., in treating viral infections) and investment in the development of new antibiotics [10].

This retrospective observational study was designed as an institutional surveillance analysis. Its objective was to describe temporal changes in microbiological testing coverage, selected antimicrobial-resistance phenotypes among clinical isolates, department-specific profiles of selected antibiotic use, and infection-related patient-safety indicators in surgical departments and the ICU of a Romanian tertiary clinical emergency hospital. Because individual-level antibiotic exposure could not be linked to individual-level resistance outcomes, the study was not designed to estimate patient-level associations or causal relationships between antibiotic consumption and antimicrobial resistance. The study involved 8141 patients who underwent antibiotic treatment over a period of 36 months in a tertiary center providing specialized surgical care across multiple departments. Demographic characteristics were available only for 46 validated HAI/*C. difficile* (CDI) cases.

Types of germs, antibiotic resistance profile, and main consumption of the class of antibiotics used in surgical wards were analyzed. The analyses were performed using routinely collected institutional data and were intended to describe temporal changes in microbiological testing, selected antimicrobial resistance phenotypes, department-specific profiles of selected antibiotics, and infection-related patient safety outcomes. Data were extracted and aggregated on a semestrial basis, generating six analytical time-points that enabled longitudinal trend analysis. Department-level aggregation was used for the selected-antibiotic Defined Daily Doses (DDD) profiles. Individual-level analysis was limited to validated healthcare-associated infection or CDI cases for which length of stay (LOS) could be verified.

## 2. Materials and Methods

### 2.1. Study Design, Setting, and Reporting Framework

This retrospective, observational, longitudinal, single-center study was conducted over six consecutive semesters (noted as S_I_ to S_VI_) from 1 January 2023 to 31 December 2025, in a tertiary clinical emergency hospital in Transylvania, Romania. The institution provides specialized surgical care across multiple departments, including general surgery, orthopedics, thoracic surgery, vascular surgery, urology, and ICU, the latter being included as a high-risk comparator because of its exposure to broad-spectrum antimicrobials, critically ill patients and healthcare-associated infection risk.

The analyses were performed using routinely collected institutional data and were intended to describe temporal changes in microbiological testing, selected antimicrobial resistance phenotypes, department-specific profiles of selected antibiotics, and infection-related patient safety outcomes. Data were extracted and aggregated on a semestrial basis, generating six analytical time-points that enabled longitudinal analysis. Department-level aggregation was used for the selected-antibiotic DDD profiles. Individual-level analysis was limited to validated healthcare-associated infection or CDI cases for which LOS could be verified.

Reporting was guided, where applicable, by the STROBE-AMS recommendations for antimicrobial-resistance and stewardship studies [19], together with the STROBE statement for observational studies [20] and the RECORD statement for studies using routinely collected health data [21]. Direct personal identifiers were removed, and datasets were processed in aggregated or anonymized form.

### 2.2. Data Sources and Integration of Data Sources

Aggregate data were extracted retrospectively from four institutional sources: the clinical microbiology laboratory database, hospital pharmacy or antimicrobial-use records, the infection prevention and control registry, and the clinical-administrative hospital information system. The microbiology database provided information on specimen type, organism identification, antimicrobial susceptibility testing results, antibiogram counts, selected resistance phenotypes, and preoperative screening results. Pharmacy records provided selected antibiotic quantities expressed as DDD [22]. The infection prevention and control registry provided validated healthcare-associated infection indicators, including CDI and postoperative wound suppuration, and demographic data regarding documented HAI/CDI cases. Clinical-administrative records provided LOS information for validated infection-related cases. Clinical microbiology records included routine diagnostic specimens collected during patient care, including urine cultures, purulent wound secretion cultures, respiratory specimens, sperm cultures, conjunctival and auricular secretions, nasal and pharyngeal exudates, and blood/catheter cultures. Diagnostic clinical cultures and screening specimens were treated as separate microbiological datasets. Clinical isolates were obtained from specimens collected for diagnostic purposes during routine care, whereas screening specimens were collected for the detection of resistant bacteria carriage or colonization according to institutional protocols. Resistance-phenotype analyses of clinical isolates did not include screening specimens unless explicitly stated.

For specimen-specific reporting, urine cultures and purulent wound secretion cultures were selected as the main descriptive categories because they were available across all six semesters. Purulent wound secretion cultures were directly related to wound-related surgical safety indicators, whereas urine cultures represented a major component of the microbiological workload and captured the main Gram-negative organisms relevant to AMR surveillance. Other clinical specimen types were retained in the overall microbiological dataset but were not tabulated separately because of their lower frequency and heterogeneous clinical origin.

### 2.3. Definitions of Resistance Phenotypes and Infection Outcomes

According to internationally accepted criteria [23], MDR was defined as non-susceptibility to at least one agent in three or more antimicrobial categories, XDR was defined as non-susceptibility to at least one agent in all but two or fewer antimicrobial categories, while PDR was defined as non-susceptibility to all agents in all relevant antimicrobial categories.

Methicillin-resistant *S. aureus* (MRSA), macrolide–lincosamide–streptogramin B resistance (MLSB), and extended-spectrum beta-lactamase (ESBL) production were defined according to the routine microbiology laboratory criteria in use during the study period.

CDIs were defined according to the institutional surveillance definition used during the study period. Postoperative wound suppuration was recorded as an infection-related surgical safety indicator.

Colonization and infection were differentiated where clinical validation permitted. For screening analyses, positive screening tests were considered carriage or colonization indicators rather than infection events.

Primary outcomes were the semester-level changes in the microbiological testing intensity index among antibiotic-treated patients, calculated as the number of antibiograms performed divided by the number of antibiotic-treated patients during each semester. This index reflects the institutional intensity of microbiological testing relative to antibiotic use and was not interpreted as direct confirmation that treatment was initiated or adjusted according to the antibiogram at the individual patient level.

Secondary outcomes included selected MDR/XDR/PDR resistance phenotype indicators, department-specific proportional profiles of selected antibiotics expressed in DDD, annual CDI counts, postoperative wound suppuration or surgical site infection counts, preoperative screening volume and positivity, etiological distribution by specimen type, and length of stay among validated HAI/CDI episodes.

### 2.4. Microbiological Procedures in Antimicrobial Susceptibility Testing

Standard microbiological identification and antimicrobial susceptibility testing (AST) were performed using validated automated systems available within the hospital microbiology laboratory. A VITEK^®^ 2 Compact automatic analyzer, a MALDI-TOF Biotyper Sirius RUO, a TC 50 thermostat, and API 20 E standardized identification kits were employed in the process. The clinical specimens analyzed included urine cultures, purulent wound secretion cultures, and other clinically indicated samples recorded during routine care. Preoperative screening specimens were analyzed separately from clinical diagnostic samples.

Susceptibility results were interpreted according to European Committee on Antimicrobial Susceptibility Testing (EUCAST) clinical breakpoints applicable for the respective year of testing [24]. Duplicate isolates obtained from the same patient within a 30-day interval were excluded from resistance trend analyses.

### 2.5. Antimicrobial Use, Preoperative Screening and HAI Analyses

Antimicrobial-use data were analyzed as department-specific proportional profiles of selected antibiotics expressed according to the WHO ATC/DDD system [22]. For each department, the absolute DDD value for each selected antibiotic was calculated, and the proportional contributions of each antibiotic to the total DDD of the selected antibiotics in that department were reported. Preoperative bacteriological screening was analyzed separately from diagnostic clinical cultures as an infection-prevention and carriage-surveillance indicator. Screening included specimens collected from several anatomical sites according to institutional protocols and was used to detect carriage with selected resistant organisms, including MRSA and resistant Gram-negative bacteria. Although MRSA was a major screening target, other resistant organisms, including MSSA, ESBL-producing Enterobacterales, and XDR/PDR Gram-negative organisms, were also recorded. Screening activity was summarized by the number of tests performed and the number and proportion of positive screening results. Positive screening results were interpreted as carriage or colonization indicators, not as clinical infection events.

HAI outcomes included CDI at hospital level and postoperative wound suppuration or surgical site infection in surgical departments. These outcomes were reported annually as descriptive counts. LOS was analyzed among validated infection-related cases, being calculated as the number of days from admission to discharge for each hospital episode.

### 2.6. Statistical Analysis

Primary data management was performed using the Excel 2016 application (Microsoft Corporation). To ensure a high degree of accuracy and complete reproducibility of the analytical flow, statistical analysis was performed using the Python programming language (version 3.12). Data manipulation and descriptive statistics were performed using the pandas and NumPy packages. Data distribution of demographic characteristics and clinical variables (e.g., LOS, distribution by ward) was generated using the matplotlib and seaborn libraries. The Cochran–Armitage linear trend test for proportions was applied to assess the presence and direction of monotonic trends across the six consecutive semesters, specifically for the microbiologically guided prescribing rate and MDR/XDR pathogen isolation rates, only when strict appropriate denominators were available (e.g., microbiological testing coverage). Fisher’s exact test was used when comparing the phenotypic resistance profiles between the initial and final study years, given the small absolute case numbers for certain pathogens. The odds ratio was calculated as a measure of effect size for dichotomized comparisons. Spearman’s rank correlation coefficient was calculated as an exploratory measure of the association between total antibiogram volume per semester and the XDR *P. aeruginosa* number of isolates, taking into consideration the limited number of data points. It therefore denoted that non-significant findings were considered inconclusive rather than evidence towards the absence of an association. All tests were two-tailed, a significance level of α = 0.05 being applied throughout.

### 2.7. Ethical Considerations

This study was conducted in full compliance with the ethical principles of the revised Declaration of Helsinki and was approved by the hospital’s Ethics Committee (reference no. 40/27.05.2026). As this retrospective study was based exclusively on anonymized aggregate institutional records collected as part of routine clinical care and mandatory surveillance, involved no direct patient contact, and did not include identifiable individual-level patient information, individual patient informed consent was formally waived out. All data handling procedures complied with applicable data protection legislation, including Regulation (EU) 2016/679 (GDPR).

## 3. Results

### 3.1. Analytical Datasets

Study data covered 36 consecutive months in 2023–2025 and were extracted from routine institutional records. Across the six study semesters, the institutional dataset included 8141 antibiotic-treated patient records and 1892 laboratory-reported antibiogram events. Results are presented according to the level at which each dataset was available as summarized in Table 1.

### 3.2. Microbiological Testing Coverage Among Antibiotic-Treated Patient Records

The hospital-wide microbiological testing intensity index increased over the study period. The number of laboratory-reported antibiograms relative to antibiotic-treated patient records increased from 18.05% in S_I_ to 28.20% in S_VI_ (Table 2). The overall Cochran–Armitage trend test across the six semesters was statistically significant (*p* < 0.001). Because the numerator consisted of laboratory-reported antibiogram events, this outcome was reported as an institutional microbiological testing intensity index rather than as patient-level confirmation of antibiogram-guided prescribing.

### 3.3. Selected Resistance-Phenotype Indicators

Selected resistance phenotypes showed heterogeneous semester-level patterns when expressed as proportions of species-specific clinical isolates. *E. coli* MDR was highest in S_II_, at 10/136 isolates (7.4%; 95% CI 4.0–13.0), and was not detected in S_VI_, 0/122 isolates (0.0%; 95% CI 0.0–3.1). *S. aureus* clinical isolates with combined methicillin resistance and macrolide–lincosamide–streptogramin B resistance showed a decrease in the MRSA+MLSB phenotype among purulent secretion isolates, from 11/36 isolates in S_II_ to 1/43 isolates in S_IV_, and no MRSA+MLSB clinical isolates being recorded in 2025. In S_VI_, XDR/PDR *P. aeruginosa* was identified as a local laboratory surveillance signal. Among clinical diagnostic isolates, four XDR/PDR *P. aeruginosa* isolates were detected among 25 clinical *P. aeruginosa* isolates, corresponding to 16.0%. These clinical detections originated from different specimen categories, including purulent secretion cultures, a respiratory specimen, and a blood/catheter-related culture. One additional XDR/PDR *P. aeruginosa* detection was recorded through screening and was analyzed separately as a carriage/colonization indicator, not as a clinical infection isolate. Other identified phenotypes included *E. coli* XDR, *E. coli* PDR, *Klebsiella pn*. MDR, *P. aeruginosa* MDR, *S. aureus* MRSA and *S. aureus* XDR. Because of the low absolute number of isolates and the absence of molecular typing, most findings were interpreted descriptively. Results were significant (*p* < 0.001) for *S. aureus* MRSA+MLSB. Regarding *E. coli* MDR, results were significant (*p* < 0.001) as overall between-semester difference, not as a general trend. For *P. aeruginosa* XDR/PDR, results were significant (*p* = 0.001) as a descriptive surveillance indicator, not as evidence of confirmed epidemiological re-emergence or an outbreak (Table 3).

### 3.4. Exploratory Association Between Testing Volume and P. aeruginosa MDR/XDR Detection

A Spearman’s test found no correlation between the number of antibiograms per semester and the *P. aeruginosa* MDR/XDR number of isolates. Given the aggregated data collection in only six semesters, the analysis was regarded as exploratory and inconclusive (Table 4).

### 3.5. Department-Specific Proportional Profiles of Selected Antibiotics

Antimicrobial-use analyses focused on four stewardship-relevant selected antibiotics: cefuroxime, amoxicillin–clavulanate, ceftriaxone, and meropenem. These agents were selected to represent common surgical/perioperative use, empirical broad-spectrum therapy, third-generation cephalosporin exposure, and reserve carbapenem use. The results therefore describe department-specific profiles of selected agents rather than total antimicrobial consumption. The total amount of active substance used was converted from g/100 patient-days into DDD units using the WHO assigned DDD for that substance and route in the case of the four selected antibiotics. DDD were standardized against the total surgical workload across the 2023–2025 period (19,915 patient-days). In the predefined analytical subset comprising the selected surgical departments and ICU comparator, the pooled selected-antibiotic profile comprised 28,790.9 DDD. Amoxicillin–clavulanate accounted for the largest share, with 11,832.9 DDD (41.1%), followed by cefuroxime with 9111.0 DDD (31.6%), ceftriaxone with 6010.0 DDD (20.9%), and meropenem with 1837.0 DDD (6.4%).

When ICU was excluded and only surgical departments were considered, the selected-antibiotic total was 17,552.6 DDD. In this strictly surgical subset, cefuroxime accounted for 6654.0 DDD (37.9%), amoxicillin–clavulanate for 6445.6 DDD (36.7%), ceftriaxone for 3491.0 DDD (19.9%), and meropenem for 962.0 DDD (5.5%) (Table 5).

### 3.6. Healthcare-Associated Infection Indicators

Selected infection-related patient-safety indicators were analyzed according to denominator availability. CDI was retained as a hospital-level annual descriptive count because hospital-wide patient-days, admission denominators, and CDI-testing denominators were not consistently available in the aggregate dataset. CDI cases were 13 in 2023, nine in 2024, and five in 2025. These values were not interpreted as standardized CDI incidence rates or as evidence of improved infection prevention.

For the surgical-site infection indicator, a surgical-procedure denominator was available for the whole 2023–2025 period. A total of 15 validated SSI cases were recorded among 6025 surgical procedures, corresponding to a pooled SSI rate of 0.25 infections per 100 procedures. Because annual procedure-specific denominators and risk stratification were not consistently available, this estimate was interpreted as a pooled descriptive rate rather than an annual risk-adjusted SSI incidence trend.

The semester-level distribution of validated infection-related cases showed that General Surgery and ICU together accounted for 24/46 cases (52.2%; 95% CI 36.9–67.1) across the study period (Table 6). Semester-level proportions varied from 0.0% to 77.8%, but confidence intervals were wide because of the small number of cases in several semesters. These values were interpreted descriptively and were not standardized by patient-days, admissions, surgical procedures, or case-mix characteristics.

### 3.7. Preoperative Screening Activity and Positivity

Preoperative bacteriological screening was analyzed separately from diagnostic clinical cultures. Screening volume increased from 48 tests in S_III_ to 329 tests in S_VI_. MRSA-positive screening results decreased from 10/48 tests (20.8%) in S_III_ to 2/329 tests (0.6%) in S_VI_. However, screening activity was not limited to nasal MRSA screening and included multiple anatomical sampling sites and, in some periods, target organisms other than MRSA. Therefore, the change in MRSA-positive screening results was interpreted descriptively.

### 3.8. Microbiological Sampling Patterns and Etiological Distribution by Specimen Type

Microbiological sampling patterns in the surgical departments are reflected in Table 7. Although several types of clinical specimens were processed during the study period, the specimen-specific analysis focused on urine cultures and purulent wound secretion cultures as they were consistently reported across all six semesters, represented a substantial proportion of the microbiological workload, and were clinically relevant to the surgical and patient-safety focus of the study. Other specimen types were retained only in the overall microbiological dataset.

**Table 7:** Microbiological sampling patterns in surgical departments.

Period	Laboratory Analyses			Purulent Secretion Cultures			Urine Cultures		
	Total	Surgical Departments		Total	Surgical Departments		Total	Surgical Departments	
	*n*	*n*	%	*n*	*n*	%	*n*	*n*	%
S_I_	291	84	28.87	130	75	57.69	158	6	3.80
S_II_	295	63	21.36	105	55	52.38	155	3	1.94
S_III_	323	75	23.22	88	57	64.77	196	5	2.55
S_IV_	378	103	27.25	142	88	61.97	180	2	1.11
S_V_	307	101	32.90	96	52	54.17	163	5	3.07
S_VI_	299	89	29.77	94	63	67.02	163	19	11.66

Note: The table describes sampling activity, not infection incidence.

The aggregated etiological distribution differed by specimen type. Urinary tract samples were dominated by *E. coli*, followed by *K. pneumoniae* and *Proteus mirabilis*. Purulent secretion cultures were led by *S. aureus*, followed by *E. coli* and *P. aeruginosa* (Table 8).

**Table 8:** Semestrial incidence of the leading bacterial isolates by major specimen type, 2023–2025.

Specimen Type	Rank	Bacterial Species	S_I_	S_II_	S_III_	S_IV_	S_V_	S_VI_	Cumulative Isolates (*n*)
Urine cultures	1	*E. coli*	127	118	155	129	129	113	774
Urine cultures	2	*K. pneumoniae*	17	17	17	25	18	25	116
Urine cultures	3	*Proteus mirabilis*	7	9	6	10	3	8	43
Purulent secretion cultures	1	*S. aureus*	33	36	23	43	36	22	193
Purulent secretion cultures	2	*E. coli*	18	18	14	24	12	6	92
Purulent secretion cultures	3	*P. aeruginosa*	2	9	6	16	16	17	66

### 3.9. Demographic Characteristics and Length of Stay Among Validated Infection-Related Cases

A total of 46 validated HAI/CDI cases were included in the infection-related clinical dataset. Patients were predominantly older adults, with a mean age of 61.9 years, a median age of 66.0 years, and an age range of 22–85 years; 25/46 patients (54.3%) were aged 65 years or older. Male patients accounted for 27/46 cases (58.7%). These values describe the validated infection-related cohort and were not interpreted as prevalence estimates because corresponding sex-specific, age-specific, and department-level denominators were not available.

Within the predefined surgical departments, validated infection-related cases were recorded in General Surgery, while no validated cases were recorded in Gynecology, Thoracic Surgery, Orthopedics, or Urology. ICU was retained separately as a high-acuity comparator.

Length of stay was analyzed descriptively among the same 46 validated HAI/CDI cases for which admission and discharge dates could be verified. The overall median LOS was 12.5 days. Median LOS was 15.0 days in surgical cases and 19.0 days in ICU cases. The maximum LOS was 84 days. Mean LOS values were retained for descriptive completeness, but medians were emphasized because LOS distribution was skewed by extreme values. Because no matched non-infected comparison group was available and no adjustment for confounders was performed, LOS findings were interpreted as observed hospitalization duration among patients with validated infection-related events, not as attributable excess hospitalization (Table 9).

## 4. Discussion

### 4.1. Principal Findings

This retrospective single-center institutional surveillance study integrated routinely collected microbiology, antimicrobial-use, infection-prevention, and clinical-administrative data to describe antimicrobial-resistance, stewardship-related, and infection-related patient-safety indicators in surgical departments and the ICU.

The principal findings were: an increase in the hospital-wide microbiological testing intensity index; heterogeneous semester-level proportions of selected resistance phenotypes among species-specific clinical isolates; distinct department-specific selected-antibiotic use profiles; expansion of bacteriological screening activity; and descriptive patient-safety signals including CDI counts, validated SSI cases, the distribution of validated infection-related cases in General Surgery and ICU, and observed LOS among validated HAI/CDI cases.

These findings should be interpreted as descriptive institutional surveillance signals. The increase in microbiological testing intensity may reflect greater laboratory use, improved clinician awareness, changes in culture-ordering practices, repeated testing in complex patients, or changes in the distribution of departments submitting specimens. On the background of an increased microbiological testing intensity, changes in selected resistance phenotypes may reflect true microbiological variation, but they may also be influenced by sampling intensity, specimen distribution, patient case mix, screening practices, laboratory workflow, susceptibility panels, or breakpoint interpretation over time. The use of species-specific denominators and 95% confidence intervals improves interpretability, but several resistant phenotypes had low absolute counts and wide confidence intervals. The late detection of XDR/PDR *P. aeruginosa* deserves clinical attention, but it should be interpreted cautiously. Without complete patient-level linkage, uniform duplicate-isolate exclusion, detailed ward-clustering data, and molecular typing, the study could not distinguish between sporadic detection, sampling variation, repeated isolation, localized transmission, or outbreak-related spread. CDI counts decreased descriptively during the study period, but standardized CDI incidence could not be calculated because hospital-wide patient-days, admission denominators, and CDI-testing denominators were not consistently available. SSI was reported using the available overall procedure denominator as a pooled non-risk-adjusted rate. LOS was interpreted as observed hospitalization duration among validated infection-related cases, not as attributable excess hospitalization.

### 4.2. National and European AMR and Surveillence Context

**Figure 1:**
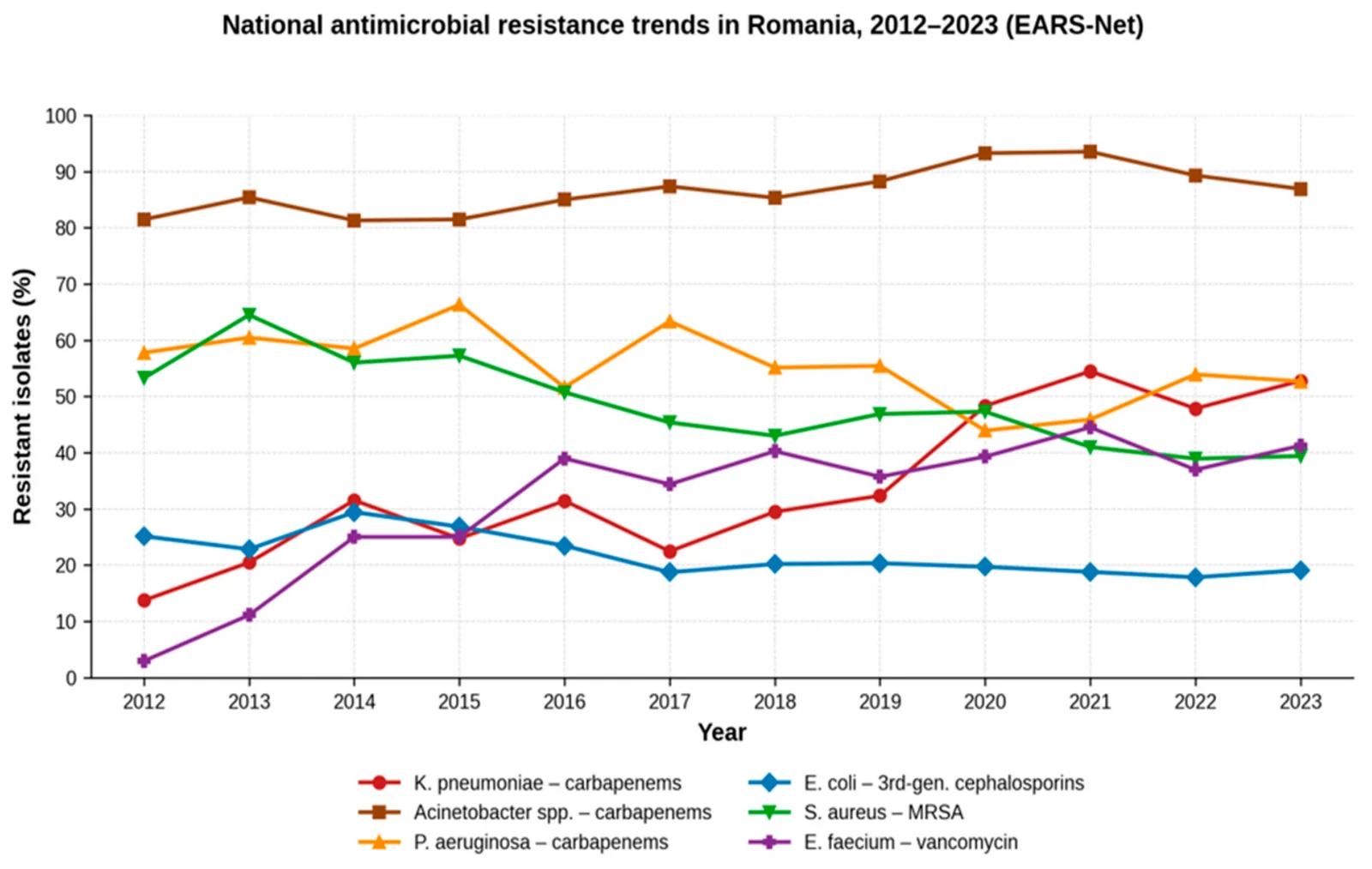
National antimicrobial resistance trends in Romania, 2012–2023, for six pathogen–antimicrobial combinations under EARS-Net surveillance. Note: Data representing the national population-weighted percentage of resistant invasive isolates reported to the ECDC Surveillance Atlas of Infectious Diseases [26] need to be interpreted with caution given the differences from the present institutional dataset in population coverage, specimen type, denominator structure, and reporting level.

Global estimates have shown that bacterial antimicrobial resistance is associated with substantial mortality worldwide [9]. Antimicrobial resistance is also a multifactorial global health problem requiring coordinated surveillance, infection prevention, antimicrobial stewardship, and policies aimed at reducing unnecessary antibiotic exposure [25]. National and international surveillance reports provide useful context for interpreting local AMR findings. National EARS-Net data retrieved from the ECDC Surveillance Atlas of Infectious Diseases [26] provided a continuous, primary-source record of resistance trends in Romania across the 2012–2023 period, as reflected in Figure 1.

The local results are compatible with the broader view that AMR surveillance requires integration of microbiological diagnostics, antimicrobial-use monitoring, infection prevention, and stewardship review [9,11,18,25,26,27,28,29,30,31]. However, the present study adds hospital-level information that is not visible in national surveillance summaries, particularly regarding specimen-specific microbiology, selected resistance signals, selected-antibiotic profiles, screening activity, and infection-related patient-safety indicators.

Romanian hospital-based studies in high-acuity and surgical settings have also reported substantial Gram-negative resistance and setting-dependent AMR patterns [32,33,34,35,36,37,38,39,40,41]. These studies provide external context for interpreting the late XDR/PDR *P. aeruginosa* signal and the department-specific antimicrobial-use profiles. They do not, however, establish that the local temporal patterns observed in the present study reflect a true epidemiological shift or a causal effect of stewardship.

### 4.3. Clinical and Stewardship Implications

The findings support the practical value of integrating microbiology, pharmacy, infection-prevention, and clinical-administrative data in routine institutional surveillance. Monitoring microbiological testing intensity may help identify changes in diagnostic activity, but future indicators should link cultures and susceptibility results to antimicrobial decisions, including escalation, de-escalation, discontinuation, and time to targeted therapy.

Department-specific selected-antibiotic profiles may help guide stewardship review of perioperative antibiotics, empirical broad-spectrum therapy, third-generation cephalosporin use, and reserve carbapenem use [42]. However, because the analysis was restricted to selected antibiotics and complete standardized exposure measures were not available for all departments, these profiles describe antimicrobial-use composition rather than total antimicrobial pressure or patient-level exposure [43].

The separation of urinary and purulent-secretion microbiology supports the development of specimen-specific cumulative antibiograms to inform local empirical-treatment recommendations [44]. The late XDR/PDR *P. aeruginosa* signal should prompt focused review of specimen source, ward clustering, repeated isolates, environmental reservoirs, prior antimicrobial exposure, and infection-prevention practices [45,46]. Screening data support continued carriage surveillance, but future protocols should clearly define eligibility criteria, target populations, sampling sites, and whether screening is universal or risk-based [47].

### 4.4. Strengths and Limitations

The main strength of this study is the integration of multiple routine institutional data sources over three consecutive years. The analysis connects microbiological activity, selected resistance phenotypes, selected antibiotic use profiles, screening activity, SSI/CDI indicators, and LOS among validated infection-related cases. This multidimensional approach is relevant for hospital-level AMR and patient-safety surveillance in surgical departments and the ICU.

Several limitations must be acknowledged. This was a single-center retrospective study using mainly aggregated routine institutional data, which limits generalizability and precludes causal inference. The study did not include a control institution, a predefined intervention period, or patient-level linkage between antimicrobial exposure, microbiological results, clinical diagnosis, comorbidities, disease severity, procedure type, ICU admission, and outcomes.

Only six semester-level observations were available for several analyses, limiting statistical power and making trend and correlation analyses exploratory.

The microbiological testing indicator was event-based. Species-specific denominators and 95% confidence intervals were added for the selected resistance-phenotype analyses. However, several resistant phenotypes had low absolute counts and wide confidence intervals, and analyses remained based on aggregated laboratory data rather than patient-level microbiological episodes. Therefore, resistance proportions were interpreted as descriptive institutional surveillance indicators.

The antimicrobial-use analysis was restricted to selected antibiotics chosen for stewardship relevance. Other antimicrobial agents were used during the study period but were not included in the selected-antibiotic profile. This selection limits interpretation of antimicrobial-use findings as total antimicrobial exposure or as a direct explanatory factor for resistance patterns. Standardized DDD/100 patient-days or DOT/100 patient-days were not consistently available for all departments [43].

Temporal changes in patient case mix, diagnostic practices, and laboratory procedures could not be fully assessed or adjusted for. Changes in surgical complexity, emergency versus elective admissions, ICU case severity, referral patterns, patient comorbidity burden, culture-ordering behavior, CDI testing, screening eligibility, specimen distribution, laboratory workflow, susceptibility testing panels, breakpoint interpretation, and reporting practices may have influenced the findings.

CDI was reported as unadjusted case counts because standardized CDI denominators were unavailable [48,49]. SSI was reported as a pooled non-risk-adjusted rate because annual procedure-specific denominators and risk adjustment were not consistently available [45,46,50]. LOS was analyzed only among validated HAI/CDI cases and was not compared with matched non-infected controls; therefore, attributable hospitalization could not be estimated. Molecular typing or whole-genome sequencing was not available, limiting the ability to distinguish sporadic detection from clonal transmission.

## 5. Conclusions

This retrospective single-center study identified institutional surveillance signals related to microbiological testing intensity, selected antimicrobial-resistance phenotypes, selected-antibiotic use profiles, screening activity, SSI indicators, CDI counts, and observed LOS among validated infection-related cases in surgical departments and the ICU. Increased microbiological testing intensity and changes in selected resistance phenotypes were observed during the study period, while the late detection of XDR/PDR *P. aeruginosa* highlighted the need for sustained microbiological surveillance and department-specific stewardship review. CDI counts decreased descriptively, and validated SSI cases corresponded to a pooled rate of 0.25 infections per 100 surgical procedures; however, these findings should be interpreted cautiously because standardized CDI denominators, annual risk-adjusted SSI rates, and patient-level exposure-outcome linkage were not available. Overall, these findings provide descriptive institutional surveillance signals and generate hypotheses for future research on antimicrobial stewardship, infection-prevention activities, and patient safety in surgical settings. Future studies should use patient-level linked datasets, standardized antimicrobial-exposure measures, standardized HAI/CDI/SSI denominators, risk adjustment for surgical procedures, clearly documented screening eligibility criteria, and molecular typing or whole-genome sequencing where appropriate.

## Figures and Tables

**Table 1:** Analytical datasets used in the results section.

Dataset	Level of Analysis	Main Denominator or Unit	Data Reported
Microbiological testing coverage	Semester level	Antibiotic-treated patient records	8141 antibiotic-treated patient records; 1892 antibiogram events
Selected resistance phenotypes	Semester level	Laboratory-reported phenotype counts	*E. coli* MDR; *P*. *aeruginosa* XDR/PDR; *S. aureus* MRSA+MLSB
Antimicrobial- use profiles	Department level	Selected-antibiotic DDD totals	General Surgery, ICU/ATI, Thoracic Surgery, Urology
HAI/CDI indicators	Semester level	Validated institutional registry counts	CDI and postoperative wound-suppuration indicators
Preoperative screening	Available periods	Screening tests and positive results	Screening volume and positivity
Length of stay	Episode level	Validated HAI/CDI episodes	46 validated infection-related cases

Note: The table summarizes analytical units and available data sources used for reporting the results.

**Table 2:** Hospital-wide microbiological testing intensity index and surgical/ICU antibiogram-event activity, 2023–2025.

Period	Antibiotic-Treated Patient Records (*n*)	Antibiogram Events (*n*)	Coverage Index (%)	95% CI (%)	Surgery Testing (*n*)	Coverage Index %	95% CI (%)
S_I_	1562	282	18.05	16.15–19.96	84	5.37	4.26–6.50
S_II_	1508	292	19.36	17.37–21.36	63	4.17	3.17–5.19
S_III_	1596	341	21.37	19.35–23.38	75	4.69	3.66–5.74
S_IV_	1313	382	29.09	26.64–31.55	103	7.84	6.39–9.30
S_V_	1077	289	26.83	24.19–29.48	101	9.37	7.64–11.12
S_VI_	1085	306	28.20	25.53–30.88	143	13.17	11.17–15.19

Note: The microbiological testing intensity index was calculated as antibiogram events divided by hospital-wide antibiotic-treated patient records in the same semester, multiplied by 100. The trend *p*-value refers to the overall Cochran–Armitage trend test across all six semesters (*p* < 0.001). Surgical/ICU values represent antibiogram events originating from surgical departments and/or ICU.

**Table 3:** Semester-level proportions of selected resistance phenotypes among species-specific isolates, 2023–2025.

Resistance Phenotype	S_I_	S_II_	S_III_	S_IV_	S_V_	S_VI_
*E. coli* MDR	1/145, 0.7% (0.0–3.8)	10/136, 7.4% (3.6–13.1)	10/169, 5.9% (2.9–10.6)	1/153, 0.7% (0.0–3.6)	1/141, 0.7% (0.0–3.9)	0/122, 0.0% (0.0–3.0)
*E. coli* XDR	0/145, 0.0% (0.0–2.5)	0/136, 0.0% (0.0–2.7)	3/169, 1.8% (0.4–5.1)	0/153, 0.0% (0.0–2.4)	1/141, 0.7% (0.0–3.9)	0/122, 0.0% (0.0–3.0)
*E. coli* PDR	0/145, 0.0% (0.0–2.5)	0/136, 0.0% (0.0–2.7)	2/169, 1.2% (0.1–4.2)	0/153, 0.0% (0.0–2.4)	0/141, 0.0% (0.0–2.6)	3/122, 2.5% (0.5–7.0)
*K. pneumoniae* MDR	1/14, 7.1% (0.2–33.9)	0/17, 0.0% (0.0–19.5)	2/17, 11.8% (1.5–36.4)	3/25, 12.0% (2.5–31.2)	0/18, 0.0% (0.0–18.5)	0/25, 0.0% (0.0–13.7)
*P. aeruginosa* XDR/PDR	2/4, 50.0% (6.8–93.2)	0/11, 0.0% (0.0–28.5)	0/7, 0.0% (0.0–41.0)	0/19, 0.0% (0.0–17.6)	0/18, 0.0% (0.0–18.5)	4/25, 16.0% (8.7–49.1)
*P. aeruginosa* MDR	1/4, 25.0% (0.6–80.6)	1/11, 9.1% (0.2–41.3)	3/7, 42.9% (9.9–81.6)	1/19, 5.3% (0.1–26.0)	1/18, 5.6% (0.1–27.3)	3/20, 15.0% (3.2–37.9)
*S. aureus* MRSA+MLSB	8/33, 24.2% (11.1–42.3)	11/36, 30.6% (16.3–48.1)	6/23, 26.1% (10.2–48.4)	2/43, 4.7% (0.6–15.8)	0/36, 0.0% (0.0–9.7)	0/22, 0.0% (0.0–15.4)
*S. aureus* MRSA	4/33, 12.1% (3.4–28.2)	6/36, 16.7% (6.4–32.8)	6/23, 26.1% (10.2–48.4)	15/43, 34.9% (21.0–50.9)	18/36, 50.0% (32.9–67.1)	12/22, 54.5% (32.2–75.6)
*S. aureus* XDR	0/33, 0.0% (0.0–10.6)	1/36, 2.8% (0.1–14.5)	0/23, 0.0% (0.0–14.8)	0/43, 0.0% (0.0–8.2)	1/36, 2.8% (0.1–14.5)	0/22, 0.0% (0.0–15.4)

Note: Values are presented as resistant phenotype counts divided by species-specific isolate denominators, *n*/*N*, %, with exact binomial 95% confidence intervals. Screening specimens were excluded from these denominators and were analyzed separately as carriage/colonization indicators. For *E. coli*, denominators were based on urinary and purulent secretion isolates. For *S. aureus*, denominators were based on purulent secretion isolates. For *P. aeruginosa*, denominators included all reported clinical isolates from urine cultures, purulent secretion cultures, respiratory specimens, otic secretions, and blood/catheter cultures, as applicable.

**Table 4:** Semester-level values used for the exploratory association analysis.

Period	S_I_	S_II_	S_III_	S_IV_	S_V_	S_VI_
Antibiogram events (*n*)	282	292	341	382	289	306
*P. aeruginosa* XDR/PDR (*n*)	2	0	0	0	0	5
*P. aeruginosa* counts	4	11	7	19	18	20

**Table 5:** Absolute and proportional DDD profiles of selected antibiotics in the surgical/ICU analytical subset, 2023–2025 aggregate.

Department	Cefuroxime, DDD (%)	Amoxicillin–Clavulanate, DDD (%)	Ceftriaxone, DDD (%)	Meropenem, DDD (%)	Total Selected DDD
General Surgery	4209.0 (33.6%)	4720.6 (37.7%)	2797.0 (22.3%)	797.0 (6.4%)	12,523.6
Thoracic Surgery	177.0 (9.2%)	1478.5 (77.1%)	204.0 (10.6%)	57.0 (3.0%)	1916.5
Vascular Surgery	478.5 (51.2%)	147.8 (15.8%)	266.0 (28.5%)	42.0 (4.5%)	934.3
Orthopedics	1729.5 (98.0%)	23.1 (1.3%)	12.0 (0.7%)	0.0 (0.0%)	1764.6
Urology	13.5 (5.0%)	14.6 (5.4%)	176.0 (65.2%)	66.0 (24.4%)	270.1
Gynecology	46.5 (32.4%)	61.0 (42.5%)	36.0 (25.1%)	0.0 (0.0%)	143.5
ICU comparator	2457.0 (21.9%)	5387.3 (47.9%)	2519.0 (22.4%)	875.0 (7.8%)	11,238.3
Total surgical/ICU subset	9111.0 (31.6%)	11,832.9 (41.1%)	6010.0 (20.9%)	1837.0 (6.4%)	28,790.9

Note: Percentages represent the internal distribution of the four selected antibiotics within each department or subset. ICU was retained as a high-acuity comparator. These values represent selected-antibiotic DDD profiles, not total antimicrobial consumption and not standardized exposure rates for all departments.

**Table 6:** Semester-level distribution of validated infection-related cases in General Surgery and ICU, 2023–2025.

Period	Total Infection- Related Cases	General Surgery	ICU	General Surgery + ICU	% of Total, 95% CI
S_I_	18	6	2	8	44.4% (21.5–69.2)
S_II_	9	6	1	7	77.8% (40.0–97.2)
S_III_	5	3	0	3	60.0% (14.7–94.7)
S_IV_	4	0	1	1	25.0% (0.6–80.6)
S_V_	1	0	0	0	0.0% (0.0–97.5)
S_VI_	9	4	1	5	55.6% (21.2–86.3)
Total	46	19	5	24	52.2% (36.9–67.1)

**Table 9:** Length of stay among validated infection-related cases, 2023–2025.

Department	Patients (*n*)	LOS (Days/ Department)	Mean LOS (Days)	Median LOS (Days)	Maximum LOS (Days)
Surgery	15	312	20.8	15.0	84
ICU	5	84	16.80	19.0	23
Infectious Diseases	8	109	13.63	13.0	23
Internal Medicine/ Gastroenterology	18	201	11.17	6.0	51
Overall validated HAI/CDI cases	46	706	15.35	12.5	84

Note: LOS was reported descriptively among validated HAI/CDI cases. Median LOS values were added because LOS distribution was skewed by extreme values. No matched non-infected comparison group was available, and no adjustment was performed for age, comorbidities, disease severity, emergency admission, procedure type, or ICU admission. Therefore, LOS values should be interpreted as observed hospitalization duration among validated infection-related cases, not as attributable excess hospitalization.

## Data Availability

Data are available from the corresponding author upon reasonable request, subject to institutional and data-protection restrictions.

## Abbreviations

The following abbreviations are used in this manuscript:

AIDS	Acquired immunodeficiency syndrome
AMR	Antimicrobial resistance
AST	Antimicrobial susceptibility testing
CDI	Clostridioides difficile infection
DDD	Defined Daily Dose
ECDC	European Centre for Disease Prevention and Control
ESBL	Extended-spectrum beta-lactamase
EUCAST	European Committee on Antimicrobial Susceptibility Testing
GLASS	Global Antimicrobial Resistance and Use Surveillance System
HAI	Healthcare-associated infection
HIV	Human immunodeficiency virus
ICU	Intensive care unit
LOS	Length of stay
MDR	Multidrug-resistant
MLSB	Macrolide–lincosamide–streptogramin B resistance
MRSA	Meticillin-resistant *Staphylococcus aureus*
MRSA+MLSB	*Staphylococcus aureus* phenotype combining methicillin resistance and macrolide–lincosamide–streptogramin B resistance
PDR	Pandrug-resistant
RECORD	REporting of studies Conducted using Observational Routinely collected health Data
S_I_	Semester I (January–June 2023)
S_II_	Semester II (July–December 2023)
S_III_	Semester III (January–June 2024)
S_IV_	Semester IV (July–December 2024)
S_V_	Semester V (January–June 2025)
S_VI_	Semester VI (July–December 2025)
SSI	Surgical site infection
STROBE	Strengthening the Reporting of Observational Studies in Epidemiology
TESSy	The European Surveillance System
WGS	Whole-genome sequencing
WHO	World Health Organization
XDR	Extensively drug-resistant
